# Supplementary material for: Supramolecular Dimer as High-Performance pH Probe: Study on the Fluorescence Properties of Halogenated Ligands in Rigid Schiff Base Complex
Source: Int J Mol Sci. 2023 May 30;24(11):9480. doi: 10.3390/ijms24119480 (PMC10253588; doi:10.3390/ijms24119480)
Supplement: Supplementary file 1 [file ijms-24-09480-s001.zip › ijms-2412323-supplementary/ijms-2412323-supplementary.docx]

**Supporting information**

Supermolecular Dimer as high-performance pH Probe: Study on the Fluorescence Properties of Halogenated Ligands in Rigid Schiff base Complex

Jiajun Xu^1^, Meifeng Huang^2^, Liang Jiao^2^, Haijun Pang^3^, Xia Wang^1^, Rui Duan^1^, and Qiong Wu^1,4,^*

^1^ Department of Chemical Science and Technology, Kunming University, Kunming, Yunnan, 650214, China.

^2^ College of Physics Science and Technology, Kunming University, Kunming, Yunnan, 650214, China.

^3^ The School of Material Science and Chemical Engineering, Harbin University of Science and Technology, Harbin, Heilongjiang, 150040, China.

^4^ Yunnan Key Laboratory of Metal-Organic Molecular Materials and Device, School of Chemistry and Chemical Engineering. Kunming University, Kunming, Yunnan, 650214, China.

* Corresponding Authors: wuqiongkm@163.com.

**Video S1.** Reversibility experiment of Zn-3,5-Cl-saldmpn.

**Video S2.** Reversibility experiment of Zn-3,5-Br-saldmpn.

**Video S3.** Reversibility experiment of Zn-saldmpn.

Figure S1: FT-IR spectra of (a) *Zn-3,5-Cl-saldmpn*, (b) *Zn-3,5-Br-saldmpn*, and (c) Zn-saldmpn; Figure S2: Absorption spectra of (a) *Zn-3,5-Cl-saldmpn*, H_2_-Cl-saldmpn, (b) *Zn-3,5-Br-saldmpn*, H_2_-Br-saldmpn, and (c) Zn-saldmpn, H_2_-saldmpn; Figure S3: The emission spectra of (a) *Zn-3,5-Cl-saldmpn*, (b) *Zn-3,5-Br-saldmpn*, and (c) *Zn-saldmpn* at different pH values (2.0–12.0); the linear relationship between the fluorescence intensity and pH (2.0–12.0) of (d) *Zn-3,5-Cl-saldmpn*, (e)*Zn-3,5-Br-saldmpn*, and (f) Zn-saldmpn; Figure S4: Fold changes of *Zn-3,5-Cl-saldmpn*, *Zn-3,5-Br-saldmpn*, and *Zn-saldmpn* in ethanol solution changed from pH 7.0 to 5.0; Figure S5: pKa of (a) *Zn-3,5-Cl-saldmpn* and (b) *Zn-3,5-Br-saldmpn*; Figure S6: Absolute PL quantum yield (QY) of *Zn-3,5-Cl-saldmpn* at pH (a) 5.0, (b) 6.0, and (c) 7.0; Figure S7: Absolute PL quantum yield (QY) of *Zn-3,5-Br-saldmpn* at pH (a) 5.0, (b) 6.0, and (c) 7.0; Figure S8: Absolute PL quantum yield (QY) of *Zn-saldmpn* at pH (a) 5.0, (b) 6.0, and (c) 7.0; Figure S9: Fluorescence lifetime of (a) *Zn-3,5-Cl-saldmpn*, (b) *Zn-3,5-Br-saldmpn*, and (c) *Zn-saldmpn* at different pH values (5.0–7.0); Figure S10: *Zn-3,5-Br-saldmpn* at different pH values (7.0–5.0) by dynamic light scattering (DLS) measurements in ethanol solution; Figure S11: Photographs of the Tyndall effect at different pH values (5.0–7.0) of (a) *Zn-3,5-Cl-saldmpn*, (b)*Zn-3,5-Br-saldmpn*, and (c) Zn-saldmpn; Figure S12: SEM images of *Zn-3,5-Br-saldmpn* at different pH values (7.0–5.0); Figure S13: The monomeric structure of Zn-χ-L, showing the selected atom type labeling. Displacement ellipsoids are drawn at the 30% probability level; Figure S14: A molecular fit of the experimental and optimized structures of *Zn-3,5-Cl-saldmpn*, *Zn-3,5-Br-saldmpn* and *Zn-saldmpn* shown in black and red, respectively; Figure S15: Interference experiment of different (a) metal ions and (b) anions on *Zn-3,5-Br-saldmpn*; Inset: Corresponding fluorescence photographs of figures a and b; Figure S16: (a) The reversibility images of *Zn-3,5-Br-saldmpn* (extracted from Video S2); The reversible response of (b)*Zn-3,5-Br-saldmpn* and (c) *Zn-saldmpn* between acidic and alkaline (blue color channel extracted from images); Figure S17: Photographs showing fluorescence color change of the *Zn-3,5-Cl-saldmpn* filter strip in response to different pH values (5.0–7.0); Figure S18: The linear relationship between blue color channel and different pH values.





**Figure S1.** FT-IR spectra of (a) Zn-3,5-Cl-saldmpn, (b) Zn-3,5-Br-saldmpn and (c) Zn-saldmpn.





**Figure S2.** Absorption spectra of (a) Zn-3,5-Cl-saldmpn, H_2_-3,5-Cl-saldmpn, (b) Zn-3,5-Br-saldmpn, H_2_-3,5-Br-saldmpn and (c) Zn-saldmpn, H_2_-saldmpn.





**Figure S3.** The emission spectra of (a) Zn-3,5-Cl-saldmpn, (b) Zn-3,5-Br-saldmpn and (c) Zn-saldmpn at different pH values (2.0-12.0); The linear relationship between the fluorescent intensity and pH values (2.0-12.0) of (d) Zn-3,5-Cl-saldmpn, (e) Zn-3,5-Br-saldmpn and (f) Zn-saldmpn.





**Figure S4.** Fold changes of Zn-3,5-Cl-saldmpn, Zn-3,5-Br-saldmpn and Zn-saldmpn in ethanol solution changed from pH values 7.0 to 5.0.





**Figure S5.** pKa of (a) Zn-3,5-Cl-saldmpn and (b) Zn-3,5-Br-saldmpn.





**Figure S6.** Absolute PL quantum yield (QY) of Zn-3,5-Cl-saldmpn at pH values (a) 5.0, (b) 6.0 and (c) 7.0**.**



 **Figure S7.** Absolute PL quantum yield (QY) of Zn-3,5-Br-saldmpn at pH values (a) 5.0, (b) 6.0 and (c) 7.0.



 **Figure S8.** Absolute PL quantum yield (QY) of Zn-saldmpn at pH values (a) 5.0, (b) 6.0 and (c) 7.0.





**Figure S9.** Fluorescence lifetime of (a) Zn-3,5-Cl-saldmpn, (b) Zn-3,5-Br-saldmpn and (c) Zn-saldmpn at different pH values (5.0-7.0)





**Figure S10.** Zn-3,5-Br-saldmpn at different pH values (7.0-5.0) by dynamic light scattering (DLS) measurements in ethanol solution.





**Figure S11.** Photographs of the Tyndall effect at different pH values (5.0-7.0) of (a) Zn-3,5-Cl-saldmpn, (b) Zn-3,5-Br-saldmpn and (c) Zn-saldmpn.





**Figure S12.** SEM images of Zn-3,5-Br-saldmpn at different pH values (7.0-5.0).


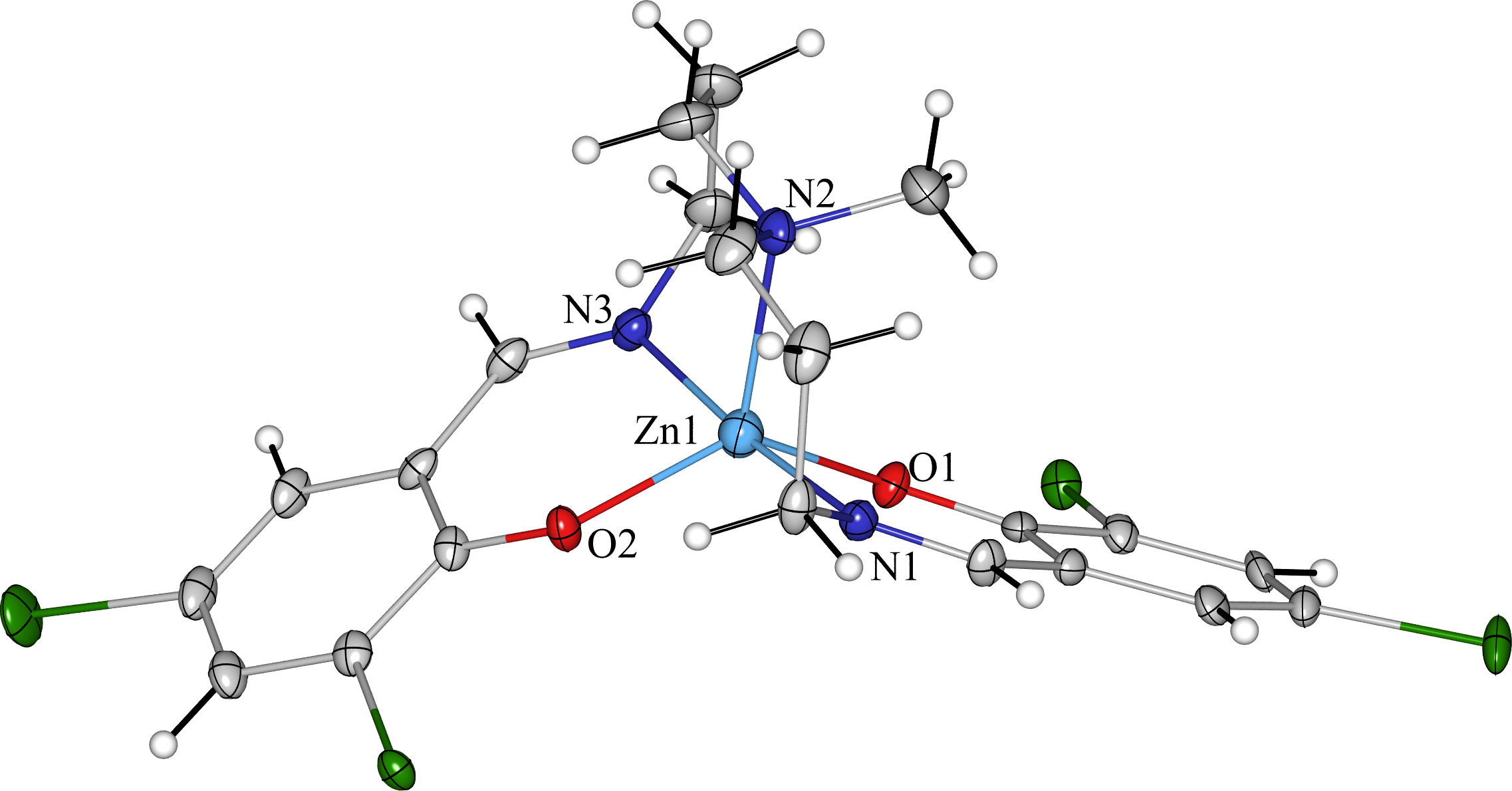


**Figure S13.** The monomeric structure of Zn-χ-L, showing the selected atom type labeling. Displacement ellipsoids are drawn at the 30% probability level.


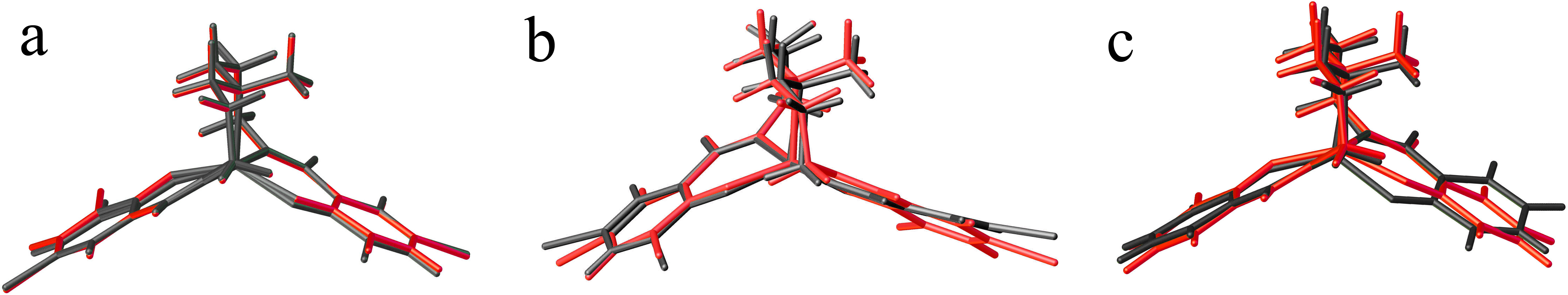


**Figure S14.** A molecular fit of the experimental and optimized structures of (a) Zn-3,5-Cl-saldmpn, (b) Zn-3,5-Br-saldmpn and (c) Zn-saldmpn shown in black and red, respectively.





**Figure S15.** Interference experiment of different (a) metal ions and (b) anions on Zn-3,5-Br-saldmpn; Inset: Corresponding fluorescence photographs of figures a and b.





**Figure S16.** (a) The reversibility images of Zn-3,5-Br-saldmpn (extracted from video 2); The reversible response of (b) Zn-3,5-Br-saldmpn and (c) Zn-saldmpn between acidic and alkaline (Blue color channel extracted from images).


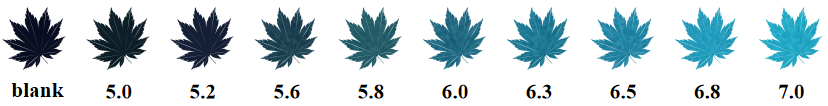


**Figure S17.** Photographs showing fluorescence color change of the Zn-3,5-Cl-saldmpn filter strip response to different pH values (5.0-7.0).





**Figure S18.** The linear relationship between blue color channel and different pH values.

**Table S1.** Crystallographic data and structure refinement for compound Zn-3,5-Cl-saldmpn and Zn-3,5-Br-saldmpn

| Identification code | *Zn-3,5-Cl-saldmpn* | *Zn-3,5-Br-saldmpn* |
| --- | --- | --- |
| Empirical formula | C_42_H_42_Cl_8_N_6_O_4_Zn_2_ | C_42_H_42_Br_8_N_6_O_4_Zn_2_ |
| Formula weight | 1109.15 | 1464.83 |
| Temperature/K | 170.0 | 151.0 |
| Crystal system | monoclinic | monoclinic |
| Space group | P2_1_/c | P2_1_/c |
| a/Å | 19.7662(5) | 20.0716(5) |
| b/Å | 12.8960(3) | 13.1084(4) |
| c/Å | 20.0635(5) | 20.2875(6) |
| α/° | 90 | 90 |
| β/° | 114.5260(10) | 114.7410(10) |
| γ/° | 90 | 90 |
| Volume/Å^3^ | 4652.8(2) | 4847.8(2) |
| Z | 4 | 4 |
| ρ_calc_g/cm^3^ | 1.583 | 2.007 |
| μ/mm^‑1^ | 1.539 | 7.630 |
| F(000) | 2256.0 | 2832.0 |
| Crystal size/mm^3^ | 0.15 × 0.14 × 0.12 | 0.2 × 0.12 × 0.12 |
| Radiation | MoKα (λ = 0.71073) | MoKα (λ = 0.71073) |
| 2Θ range for data collection/° | 3.868 to 52.788 | 3.814 to 52.792 |
| Index ranges | -24 ≤ h ≤ 24, -16 ≤ k ≤ 16, -25 ≤ l ≤ 25 | -24 ≤ h ≤ 25, -15 ≤ k ≤ 16, -25 ≤ l ≤ 22 |
| Reflections collected | 35384 | 37115 |
| Independent reflections | 9488 [R_int_ = 0.0484, R_sigma_ = 0.0478] | 9907 [R_int_ = 0.0838, R_sigma_ = 0.0833] |
| Data/restraints/parameters | 9488/0/589 | 9907/0/589 |
| Goodness-of-fit on F^2^ | 1.065 | 1.002 |
